# Clinical and Dosimetric Implications of Intrafractional Cylinder Movement During Vaginal Cuff Brachytherapy

**DOI:** 10.7759/cureus.6165

**Published:** 2019-11-15

**Authors:** Benjamin E Onderdonk, Tianming Wu, Hania Al-Hallaq, Christina H Son, Joseph Waller, Yasmin Hasan

**Affiliations:** 1 Department of Radiation and Cellular Oncology, University of Chicago Medical Center, Chicago, USA

**Keywords:** vaginal cuff, cylinder, brachytherapy, movement, intrafraction motion

## Abstract

Introduction

To quantify the dosimetric and clinical effects of intrafractional cylinder movement in patients receiving high-dose-rate vaginal cuff brachytherapy (VBT) without a formal immobilization device and the implication of motion on institutional clinical outcomes.

Methods

From 2013-2018, 119 patients were treated with VBT with no formal immobilization device at a single institution. As a quality assessment study, pre-and post-cylinder brachytherapy kilovoltage (kV) images were acquired for 37 fractions in nine consecutive patients who underwent VBT and clinical care representative of institutional practice standards. The D90 and D90 EqD2 were calculated according to each patient’s average intrafractional movement throughout the treatment course. The D2cc for organs-at-risk (OARs) were also re-evaluated following the simulated movements. The survival outcomes and toxicity were recorded from the 119 patients. Toxicity was graded as per Common Terminology Criteria for Adverse Events (CTCAE) version 4.0.

Results

The measured mean ± standard deviation movement was 5.0 mm ± 3.5, with 62% moving caudad. The D90 from each patient’s maximum and average movements were lower than the pre-planned doses: 71%, and 89%, respectively. The doses to the OARs were lower than the pre-planned doses. After a median follow-up of 20 months, there were three local recurrences with a median time of 14.5 months (range: 10-31). There were two acute grade 3+ toxicities and one late grade 3+ toxicity. There was a moderate correlation (r = 0.40) between body mass index (BMI) and intrafraction movement with caudad being more common in smaller BMIs (p = 0.0216).

Conclusions

Intrafractional vaginal cylinder movement without a table fixation device is about 5.0 mm, with the majority of movements moving caudad. While institutional outcomes suggest that local control may not be compromised, consideration of more formal immobilization devices is warranted, especially for those patients with lower BMIs.

## Introduction

Vaginal cuff brachytherapy (VBT) has been widely accepted as an adjuvant radiation modality for gynecologic malignancies, with a recently published survey of practitioners demonstrating a wide heterogeneity of the methods, dose, and fractionation [1]. In addition, the most recent American Brachytherapy Society guidelines give no clear consensus regarding a table fixation device for further immobilization of the cylinder [2]. Also, the vaginal cuff has demonstrated considerable interfraction movement between external beam radiation fractions [3]. Given these findings, and since each brachytherapy fraction is several minutes in duration, there is a considerable concern that the cylinder may move by the end of the treatment and affect the dose distribution to the target and organs-at-risk (OARs). There are currently no reports on the possible intrafraction movement during a VBT fraction and the subsequent doses to the target and organs-at-risk (OARs).

At our institution, VBT is performed without a formal table fixation device and only padding material between the cylinder and the patient’s thighs. This present study aimed to quantify the intrafractional movement of the cylinder in patients receiving VBT for gynecologic malignancies, identify correlates with the degree of movement, and calculate the doses to the target and OARs for each of these simulated movements. In addition, clinical outcomes in all patients are reported to determine the possible effect of intrafractional motion on vaginal recurrence rates as compared to those reported in the literature.

## Materials and methods

From April 2013 to April 2018, 119 patients with gynecologic malignancies underwent 472 VBT fractions with no formal immobilization device (IRB 09123B). The clinical cohort of patients consisted of the first 110 patients. A sample of the most recent nine consecutive patients comprised the dosimetric cohort. The simulated dosimetric values were generated from the intrafraction movement in the dosimetric cohort. Patient and treatment characteristics were recorded for each cohort, and the survival outcomes were recorded from all patients. Toxicities were graded as per Common Terminology Criteria for Adverse Events (CTCAE) Version 4.0. Acute toxicities were defined as toxicities occurring during treatment and up to 90 days of follow-up. Late toxicity was defined as those occurring greater than 90 days after treatment. These toxicities were recorded during each patient’s weekly on-treatment visits (during external beam radiation therapy (EBRT) or VBT) and regular follow-up visits.

**Figure 1:**
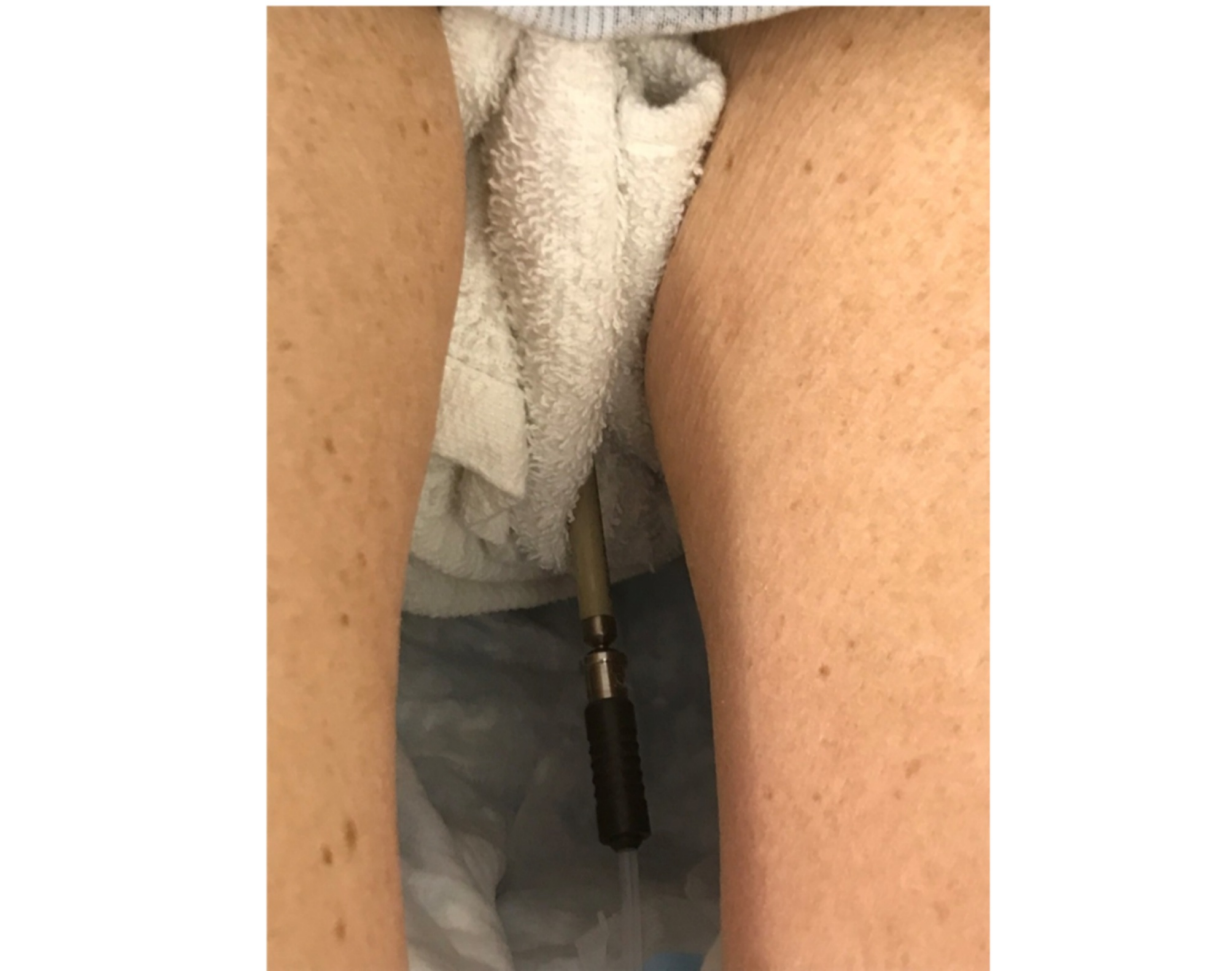
An example vaginal cuff brachytherapy setup

From the dosimetric cohort, there were 37 fractions of VBT. After cylinder placement, a folded towel padding around the central tandem was used to separate the device from the patients’ legs and provide stability of the device against the patient’s perineum (Figure 1). A dummy wire was placed into the central tandem and pre-brachytherapy (VBT) anterior-posterior (AP) kilovoltage (kV) images were acquired. The dummy wire was then removed, and the afterloader catheter was then connected and treatment was delivered. After treatment delivery, the afterloader catheter was disconnected and the dummy wire was then re-inserted. Post-VBT AP kV images were then acquired.

The superior aspect of the dummy wire was measured to the superior pubic symphysis in each of these images and intrafraction motion was defined as the difference between these measurements. Each patients’ maximum intrafraction movement and average movements throughout the treatment course were recorded. Patient and treatment characteristics were recorded to identify associations between intrafraction movements. These characteristics included: patient body mass index (BMI), distance from the superior aspect of the vaginal cylinder to the superior pubic ramus/pubic symphysis (pre-VBT distance), cylinder diameter, and treatment duration (individual fraction, average, and total).

To ease the intrafraction movement dosimetric calculations, a 2 mm isovolumetric shell was created by expanding the target volume (vaginal surface/cylinder surface) by 2 mm into the surrounding soft tissue. The absolute D90 and biological D90 EqD2 were calculated to the volume of this shell according to each patient’s maximum and average intrafractional movement throughout the treatment course. The absolute D2cc and biological D2cc EqD2 for the bladder, rectum, and sigmoid colon were also re-evaluated following these simulated movements.

Statistical analyses were performed with JMP Version 14.0 (Marlow, Buckinghamshire, England). From the dosimetric cohort, a bivariate, one-way analysis of variance (ANOVA) and a 2-tailed t-test were performed to correlate intrafraction movement with patient and treatment characteristics: age, BMI, pre-BT distance, cylinder diameter, individual fraction duration, and total treatment duration. The differences in patient and treatment characteristics between the cohorts were examined using chi-square for the categorical variables and ANOVA for the continuous variables.

From the entire patient cohort, the Kaplan-Meier method was used for calculating recurrence rates and for survival analysis. Follow-up time was calculated in months from the date of the primary diagnosis until the date of the last follow-up. Local recurrences were defined as recurrences in the vaginal cuff. Regional recurrences were defined as recurrences within the pelvis outside of the vaginal cuff. Survival was calculated in months from the date of diagnosis until the date of local recurrence for local recurrence-free survival/local control (LC), any recurrence or death by malignancy for disease-specific survival (DFS), and death by any cause for overall survival (OS). Due to the wide heterogeneity in tumor histology, and prescription doses, these factors were not included in the statistical analyses. All of the other patient data were included in the analyses, and patients who were alive were censored at the date of the last contact. The log-rank test was performed to compare survival between different categories of patients and their characteristics. A p-value <0.05 was considered significant. The characteristics that were considered statistically significant (p-value <0.05) or demonstrated a trend for significance (p-value < 0.10) were included in a Cox proportional hazard model.

## Results

The 119 patients in the dosimetric and clinical cohorts were comparable, with endometrioid adenocarcinoma being the most frequent histology: 56% and 52%, respectively. Also, the median pre-operative tumor sizes were 4.2 cm and 3.5 cm, respectively. In addition, the majority of patients were Stage I (56% and 55%, respectively) and were treated in the setting of primary adjuvant treatment (89% and 92%, respectively). The only difference between the cohorts was the presence of cervical stromal invasion (p = 0.019), which was present in 30 patients in the clinical cohort and none in the dosimetric cohort. For both the clinical and dosimetric cohorts, a summary of the patient characteristics are listed in Table 1.

**Table 1:** Patient characteristics LVSI - Lymphovascular Space Invasion, MMI - Myometrial Invasion, CM - centimeter, ANOVA - Analysis of Variance, N - Number.

Factors	Dosimetric Cohort (N = 9)	Clinical Cohort (N = 110)	Chi Square or ANOVA
Median Age at Initial Diagnosis (range)	60 (46-69)	63 (41 - 87)	P = 0.26 (ANOVA)
Histology			
Endometrioid Adenocarcinoma	5 (56%)	57 (52%)	P = 0.40 (Chi Square)
Low Grade	3 (60%)	11 (19%)	
Moderate Grade	1 (20%)	28 (49%)	
High Grade	1 (20%)	18 (32%)	
Endometrial Serous	1 (11%)	24 (22%)	
Carcinosarcoma	1 (11%)	8 (7%)	
Endometrial Stromal Sarcoma	-	2 (2%)	
Endometrial Clear Cell	-	9 (8%)	
Fallopian/Ovarian Serous Adenocarcinoma	2 (22%)	3 (3%)	
Cervical Carcinoma	-	3 (3%)	
Vaginal Carcinoma	-	3 (3%)	
Other	-	1 (1%)	
Pathologic Features			
Median Tumor Size (cm)	4.2 (2.0 - 10.6)	3.5 (0.1 - 16.5)	P = 0.57 (ANOVA)
Positive Margin	1 (11%)	4 (4%)	P = 0.51 (Chi Square)
LVSI	2 (22%)	39 (35%)	P = 0.27 (Chi Square)
Cervical Stromal Invasion (if Endometrial)	0 (0%)	30 (30%)	P = 0.019 (Chi Square)
MMI (if Endometrial)			P = 0.63 (Chi Square)
<50%	3 (43%)	44 (44%)	
>50%	3 (43%)	50 (50%)	
100%	1 (14%)	6 (6%)	
Stage			P = 0.30 (Chi Square)
Stage I	5 (56%)	60 (55%)	
Stage II	-	16 (15%)	
Stage III	4 (44%)	31 (28%)	
Stage IV	-	3 (3%)	
Treated at Initial Diagnosis	7 (78%)	101 (92%)	P = 0.22 (Chi Square)
Treated at Recurrence	2 (22%)	9 (8%)	

The treatment characteristics for the dosimetric cohort, which consisted of the median (range) surface prescription dose, the fractional and the total treatment D90 EqD2 to the 2 mm shell were 30 Gy (12-33), 6.2 Gy (4.6-7.4), and 28.8 Gy (18.5-36.8), respectively. The prescribed median BT dose to the vaginal mucosa was 30 Gy in five fractions for an EqD2 of 40 Gy. A summary of treatment characteristics is listed in Table 2.

**Table 2:** Treatment characteristics VBT - Vaginal Brachytherapy, EqD2 - Equivalent Dose in 2 Gy/fraction, Gy - Gray, WP - Whole Pelvic Radiation, EF - Extended Field Radiation, Carbo - Carboplatin, Taxol - Paclitaxel, EBRT - External Beam Radiotherapy, ANOVA - Analysis of Variance.

Factors	Dosimetric Cohort (N = 9)	Clinical Cohort (N = 110)	Chi Square or ANOVA
Surgical Resection Prior to VBT	9 (100%)	109 (99%)	P = 0.69 (Chi Square)
Median VBT EqD2 Dose (Gy)	40 (16 - 45.65)	29.75 (16 - 45.65)	P = 0.50 (ANOVA)
Median VBT Fractions	5 (2 - 5)	4 (2 - 6)	P = 0.48 (Chi Square)
Average Treatment Time (seconds)	409 (237 - 623)	-	-
Median Size of Cylinder (cm)	3.5 (3.0 - 4.0)	3.5 (2.5 - 4.5)	P = 0.66 (Chi Square)
Median Total EqD2 Dose to the Vaginal Cuff (EBRT + VBT) (Gy)	45.65 (40 - 69.25)	68.25 (24 - 88.5)	P = 0.49 (ANOVA)
EBRT	4 (44%)	62 (56%)	P = 0.49 (Chi Square)
EBRT Dose (Gy)	45	45 (45 - 50.4)	P = 0.41 (ANOVA)
Median EBRT Fractions	25	25 (25 - 28)	P = 0.48 (ANOVA)
Whole Pelvis	3 (75%)	47 (76%)	P = 0.18 (Chi Square)
Extended Field	1 (25%)	15 (24%)	
Concurrent Chemotherapy			P = 0.48 (Chi Square)
WP: Weekly Cis-RT	-	3 (6%)	
WP: Other Concurrent Chemotherapy	-	1 (2%)	
WP: Concurrent + Adjuvant Chemotherapy	-	4 (9%)	
EF: Weekly Cis-RT	1 (100%)	2 (13%)	
EF: Concurrent + Adjuvant Chemotherapy	-	1 (7%)	
Adjuvant Chemotherapy	3 (33%)	51 (46%)	P = 0.48 (Chi Square)
Carbo/Taxol	3 (100%)	47 (92%)	
Other Chemotherapy	-	4 (8%)	
Median Chemo Cycles	6 (4 - 6)	4 (1 - 6)	

**Figure 2:**
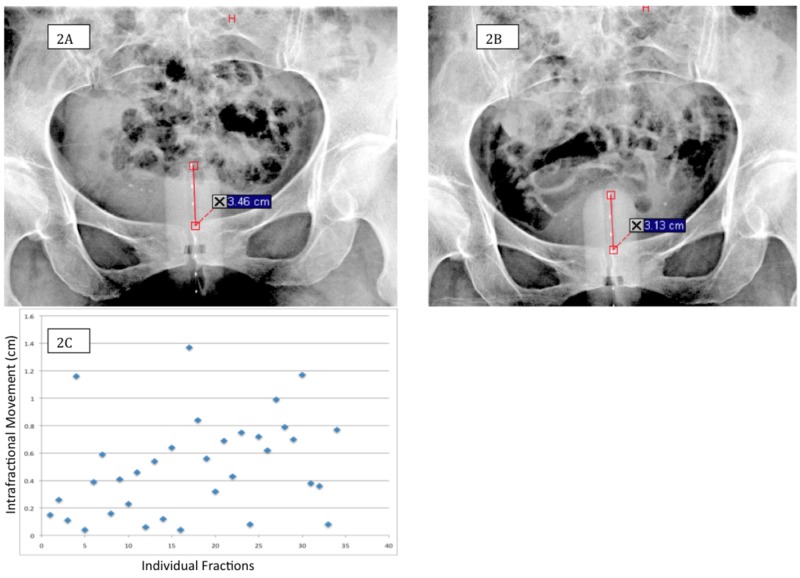
Intrafractional movement (Figure 2A): Pre-treatment anterior-posterior kilovoltage image of a patient demonstrates a distance (3.46 centimeter) measured from the superior edge of the pubic symphysis to the middle of the superior bead in the radio-opaque dummy wire. (Figure 2B): Post-treatment kilovoltage image of the same patient in Figure 1A demonstrates the post-treatment distance (3.13 centimeter). The difference reflects 0.33 cm of intrafraction motion. (Figure 2C): The mean ± standard deviation absolute value movement for the 34 fractions was 5.0 millimeter ± 3.5. CM - Centimeter

**Figure 3:**
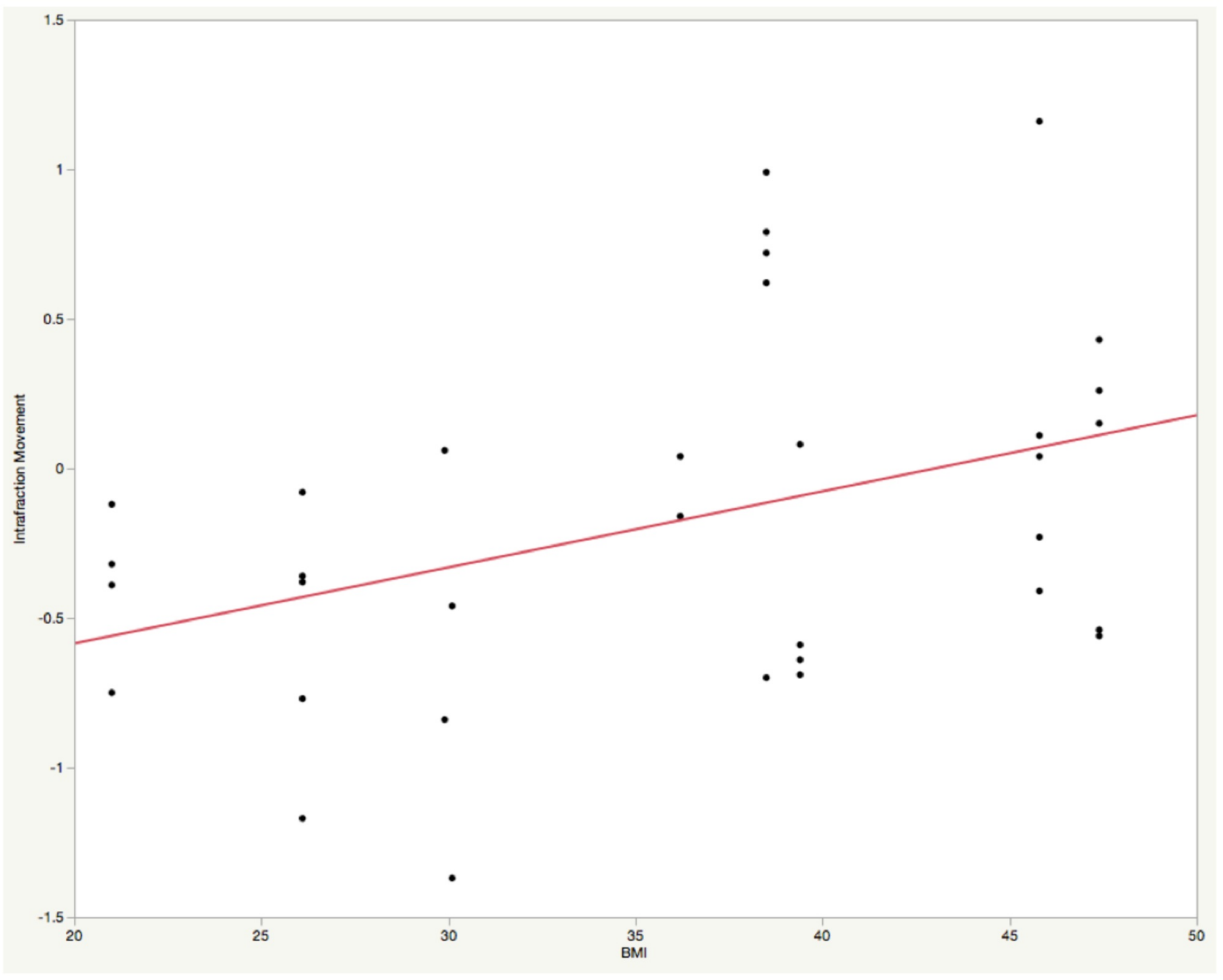
Intrafraction movement by body mass index The scatter plot depicts the bivariate fit of intrafractional movement (negative numbers representing caudad movement and positive numbers representing cephalad movements) by body mass index (p = 0.0216). There was a moderate correlation between body mass index and intrafractional movement (r = 0.40). BMI - Body Mass Index

Of the 37 fractions of VBT in the dosimetric cohort, 34 of these treatments had pre-BT and post-BT AP kV images. Example pre-treatment and post-treatment kV images are shown below in Figures 2A-2B. Twenty-one (62%) of these movements were caudad while 13 (32%) demonstrated cephalad movements toward the patient’s vaginal apex. The absolute value mean ± standard deviation movement for the 34 fractions was 5.0 mm ± 3.5, with the maximum movement being a caudad movement of 1.37 cm (Figure 2C). Bivariate, one-way ANOVA was used to correlate intrafraction motion with various treatment characteristics and demonstrated that the intrafraction movement did not correlate with patient age (p = 0.19), pre-treatment distance (p = 0.11), cylinder diameter (p = 0.15), individual fraction duration (p = 0.28), or total treatment duration (p = 0.28). However, BMI was significantly associated with caudad or cephalad movement (p = 0.0216). There was a moderate correlation for caudad movement as BMI decreased and cephalad movement as BMI increased (r = 0.40, Figure 3).

**Figure 4:**
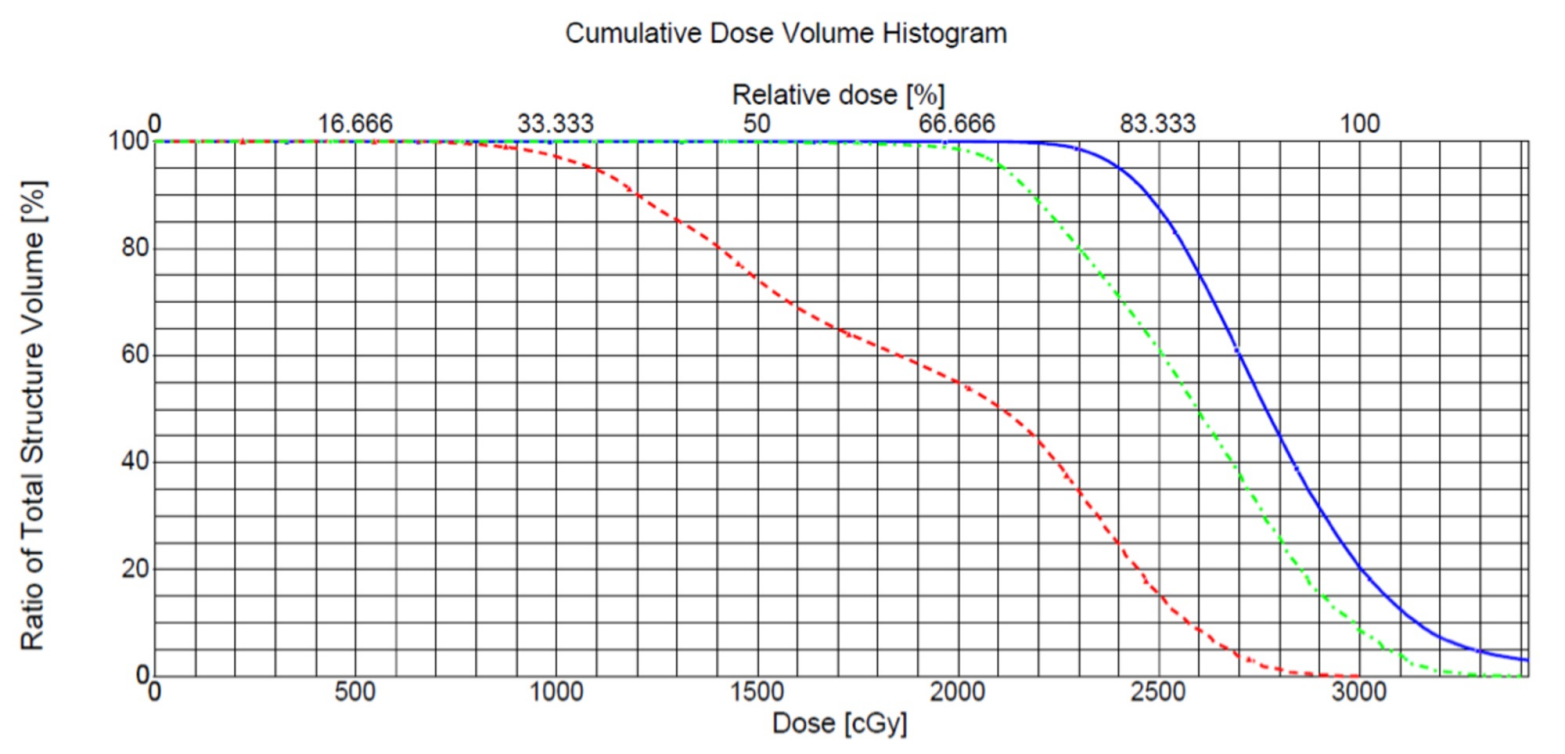
An example dose volume histogram An example dose-volume histogram (DVH) from a patient represents the prescription dose to the 2-millimeter shell (blue), the dose to the shell after the patient’s average movement of 4 millimeter (green), and dose to the shell after the maximum movement of 12 millimeter (red). cGy - Centigray

To estimate the effect of intrafraction movement on patient dosimetry, the D90 to the 2 mm shell (normalized to the prescription dose) were calculated from each patient’s maximum and average movements (Figure 4). From the dosimetric cohort, the absolute D90 after the maximum and average movements were lower than the pre-planned doses: 71%, and 89%, respectively. In addition to the lower doses to the 2 mm shell, the doses to the OARs (normalized to the pre-planned doses) were lower than the pre-planned doses (Table 3).

**Table 3:** Intrafractional movement dosimetric calculations OARs - Organs at Risk, D90 - Dose received by 90% of the target volume, D2cc - Minimum dose to the highest irradiated 2 cubic centimeter of the organ at risk, EqD2 - Equivalent dose in 2 Gray/fraction, Std Dev - Standard Deviation, MM - Millimeter.

Dosimetric Calculations		D90 to the 2mm Shell	D2cc to the OARs		
			Bladder	Rectum	Sigmoid
Maximum Cylinder Movement	Absolute Dose ± (Std Dev)	70.8% ± (17.7%)	93.5% ± (8.3)	96.0% ± (12.6)	91.3% ± (43.0)
	Biological EqD2 ± (Std Dev)	68.8% ± (19.1)	90.5% ± (12.2)	94.8% ± (19.9)	94.3% ± (64.8%)
Average Cylinder Movement Throughout The Treatment Course	Absolute Dose ± (Std Dev)	89.2% ± (13.2)	97.8% ± (3.3)	97.4% ± (4.3)	92.6% ± (19.4)
	Biological EqD2 ± (Std Dev)	86.5% ± (15.8)	96.8% ± (4.8)	96.2% ± (6.2)	91.3% ± (28.3)

**Figure 5:**
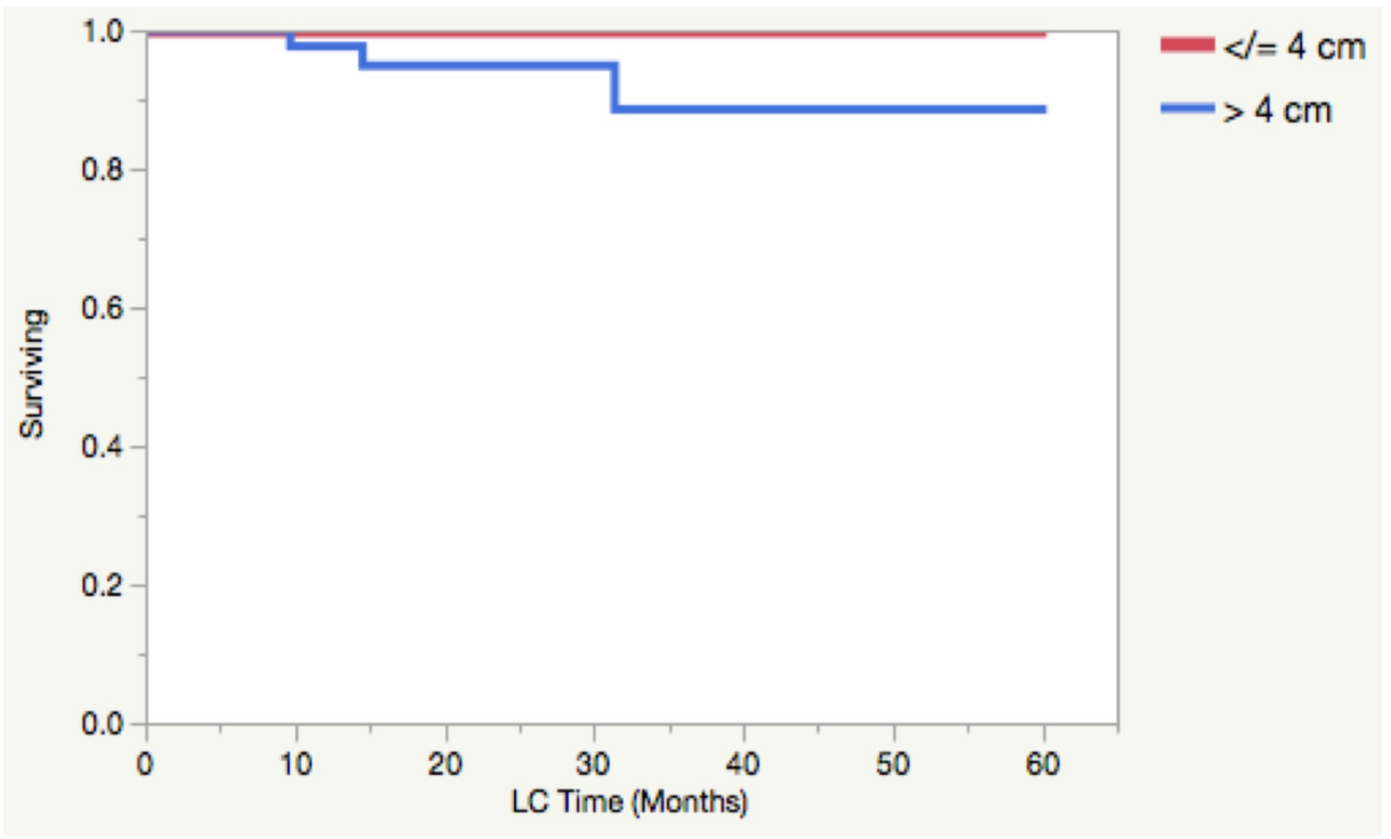
Local control by tumor size The Kaplan Meier curve depicts local control as a function of tumor size (p = 0.0523). CM - Centimeter, LC - Local Control

**Figure 6:**
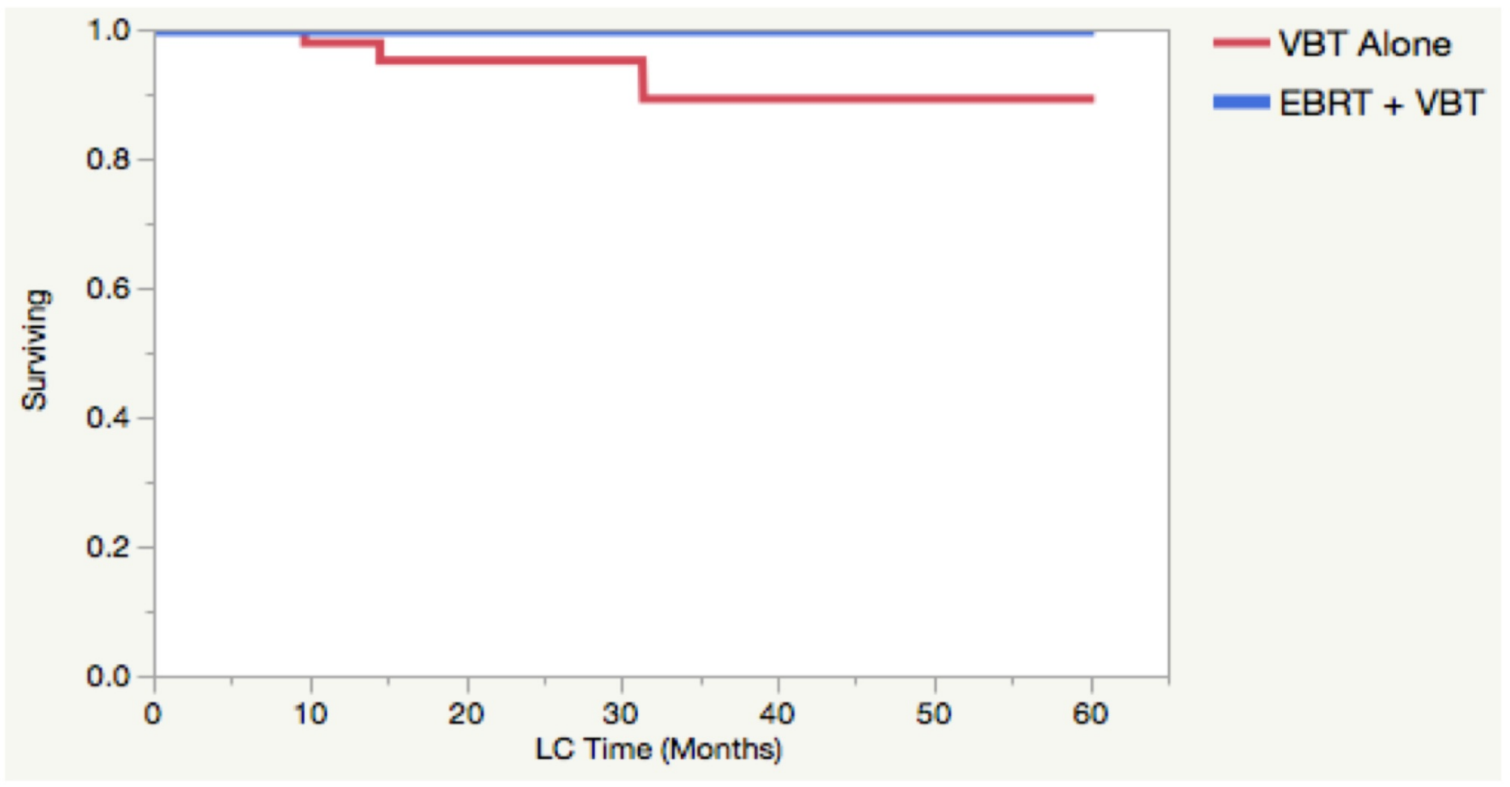
Local control by treatment The Kaplan-Meier curve depicts local control as a function of vaginal brachytherapy alone or as a boost to external beam radiation therapy (p = 0.0664). VBT - Vaginal Brachytherapy, EBRT - External Beam Radiation Therapy, LC - Local Control

Outcomes for the entire cohort of 119 patients with a median follow-up of 20 months included 19 recurrences, of which three were local (vaginal cuff). Median time for local recurrence (LR) was 14.5 months (range: 10-31). All three of the LRs occurred in patients that received an EqD2 of 40 Gy to the vaginal cuff with VBT alone, without EBRT. Of note, the two-year local failure rate in VBT alone treatment was 4.85%. However, two of the three local failures occurred in advanced-stage patients who were eligible for EBRT but did not receive it. Of patients who were candidates for VBT alone (high-intermediate risk), only one failed locally. The patient characteristics for the three local failures can be seen in Table 4. The two-year LC demonstrated a trend towards association with tumor size (100% vs 94.88 %) for tumors <4 cm and >4 cm, respectively (p = 0.0523, Figure 5), and for patients treated with VBT alone versus VBT + EBRT (p = 0.0664, Figure 6). Unfortunately, a Cox proportional hazard model was unreliable for local control due to possible multicollinearity.

**Table 4:** Local recurrences MMI - Myometrial Invasion, CSI - Cervical Stromal Invasion, LVSI - Lymphovascular Space Invasion, FIGO - International Federation of Gynecology and Obstetrics, Carbo - Carboplatin, Taxol - Paclitaxel, CM - Centimeter.

Patients	Patient and Treatment Characteristics							
	Age	Histology	MMI	CSI	LVSI	Max Dimension (cm)	FIGO Stage	Adjuvant Treatment
Patient 1	69 years	Endometrial Clear Cell Carcinoma	>50%	-	-	4.6	IVA	6 Cycles of Carbo/Taxol and Brachytherapy
Patient 2	71 years	Endometrial Adenocarcinoma (Grade 2)	>50%	-	-	6.0	IB	Brachytherapy Alone
Patient 3	80 years	Endometrial Serous Carcinoma	100%	+	+	6.5	IIIC2	6 cycles of Carbo/Taxol and Brachytherapy

Of the 19 total recurrences, five were regional recurrences (pelvic, but not vaginal cuff). The median time to regional recurrence was 15 months (range: 6 - 36), and there was a two-year regional recurrence-free survival rate of 96.8%. Distant failure occurred in 14 patients, with a median time to distant recurrence of 16 months (range: 6 - 42), and a two-year distant metastasis-free survival rate of 88.3%. The two-year disease-free survival was 84.9%, and the two-year overall survival rate was 88.1%.

The CTCAE 4.0 grade 3 or higher acute toxicities included one grade 3 genitourinary and one grade 4 mucosal toxicity. The one acute grade 4 toxicity was a labia excoriation and resolved with steroid cream. The late grade 3 or higher toxicities included only one grade 3 gastrointestinal toxicity. This patient was treated with whole pelvic EBRT + VBT boost and developed a small bowel obstruction, which required lysis of adhesions. None of these grade 3+ toxicities occurred in the dosimetric cohort.

## Discussion

Adjuvant vaginal cuff brachytherapy has been accepted as an adjuvant radiation modality for high-intermediate-risk endometrial cancer [4] and is commonly used as an additional therapy for higher stage disease [5-6] or recurrence. However, the dosing and fractionation vary between institutions with the most frequent prescription to the vaginal surface or 0.5 cm depth [7]. While there is data regarding the dosimetric effects of cylinder angulation with a table fixation device [8], the brachytherapy immobilization technique varies between institutions.

This is the first study examining the magnitude of intrafractional vaginal cylinder movement during intracavitary brachytherapy without a formal immobilization device. Without formal immobilization, the magnitude of movement is usually small, around 5.0 mm, with the majority (62%) of these moving caudad. This movement translates into a lower dose to the target, around 90% of the prescription dose throughout the patients’ treatment course (Figure 4). Of note, since patients with endometrioid-type endometrial adenocarcinoma tend to have higher BMIs [9], it is possible that a patient’s body habitus may provide some immobilization in addition to the support provided by the towel/padding (Figure 1). This study supports this notion given that BMI significantly correlated with intrafractional movement. Patients with smaller body habitus were more likely to have caudad cylinder movements while patients with larger BMIs were more likely to have cephalad cylinder movements (Figure 3, r = 0.40, p = 0.0216).

To explore the clinical impact of this movement on oncologic outcomes, the total recurrence and toxicity rates were examined from all similarly treated patients within the practice. The ability to extrapolate dosimetric cohort results to the whole group was supported by similar patient characteristics (Table 1) and by the uniform treatment method from the same radiation oncologist. This analysis demonstrated a 2.3% local failure rate at two years, which is consistent with a recent literature review demonstrating a risk for local recurrence from 0%-3.1% after vaginal cuff brachytherapy [7]. Moreover, there were low rates of grade 3 or higher toxicity with only two acute toxicities and 1 late toxicity. The late small bowel obstruction occurred in a patient who was treated with whole pelvic EBRT + VBT boost, with the location of the bowel obstruction distant from the cylinder.

While there remains variability in institutional prescription methods [1], the method in this study of prescribing to the vaginal surface as a means to lower EqD2 to the vaginal surface demonstrates a decreased risk for late vaginal stenosis [10]. After accounting for intrafraction motion in this series, the target volume (vaginal surface with a 2 mm shell) received around 90% of the prescription dose, which is supported by a report of reduced-dose VBT [11]. In that study, patients received 24 Gy in four fractions, which represents 80% of the typical VBT dose, and still demonstrated excellent local control. Since the optimum VBT dose remains unknown, the low rate of local recurrence in this study further indicates that a slightly reduced dose may be adequate.

Although these clinical outcomes were consistent with other institutional outcomes, the average movement of 5.0 mm was larger than anticipated. The local failures occurred in patients with larger (>4 cm) high or high-intermediate risk tumors: clear cell, serous, and grade 2 adenocarcinoma. Of note, these patients were all treated with VBT with or without adjuvant chemotherapy. While VBT alone remains the standard of care for high-intermediate risk endometrial cancers, with a five-year risk of vaginal cuff recurrences of less than 2% [4], the use of EBRT remains integral for high-risk endometrial cancers. Of note, on GOG 258 and patients with endometrial cancer of high-intermediate risk (PORTEC) 3, local failures were minimal when EBRT was delivered (2% and 2.1%, respectively). In contrast, in our study, two high-risk patients were treated without EBRT. In particular, one patient with stage IV clear cell carcinoma was treated with systemic therapy and palliative intent VBT. Another patient with stage IIIC2 serous carcinoma refused EBRT but elected to proceed with VBT alone and developed a local recurrence. Thus, the local recurrences in this study likely resulted from deviation from the standard of care (Table 3), and these patients would likely have derived benefit from the addition of EBRT.

Furthermore, pre-operative tumor size (Figure 5) and VBT alone (Figure 6) may be possible additional risk factors for local failure. It is unclear if the tumor size is another surrogate for poor tumor biology, but a recent large retrospective study demonstrated tumor size to be an additional risk factor in low-risk endometrial cancer [12]. While these observations regarding tumor and patient characteristics remain hypothesis-generating for local control, further studies with more patients and event outcomes are needed to confirm their clinical relevance.

Some limitations to this current study include the manner in which the dose to the target was assessed. Without objective soft-tissue assessment, the vaginal cuff may collapse onto the cylinder during caudad movement, resulting in higher doses than calculated (Figure 4). Thus, our estimate of the reduced dose to the target may be conservative for patients that receive VBT as a boost to EBRT. Also, AP kV images could not account for the possibility of angulation, which correlates with increased bladder and rectal doses, respectively [13-15], and a further decrease in dose to the mucosa from air pocket formation [16]. While these are all possibilities during intrafraction movements, pre-VBT and post-VBT cone-beam CT scans would correct for these limitations. Nonetheless, the efficacy and toxicity in this study match those previously reported.

Since these findings were identified, the department practice has moved towards more formal immobilization to limit the potential for undertreatment in select patients. Similar to other retrospective reviews, tumor size may be explored further as a potential adverse feature.

## Conclusions

This study is the first of its kind to demonstrate the magnitude of intrafractional vaginal cuff brachytherapy cylinder movement without a formal immobilization device. The average movement was 5.0 mm, and the direction of movement correlated with patients’ BMI. Moreover, intrafraction movement resulted in the vaginal target receiving only 90% of the prescribed dose and still demonstrated adequate local control. This work highlights the importance of formal immobilization during VBT in order to minimize caudad motion and potential undertreatment, especially in those patients with lower BMI.
